# YES Oklahoma: Building Pathways into Cancer Research and Public Health for Indigenous High School Students

**DOI:** 10.1007/s13187-026-02905-1

**Published:** 2026-05-22

**Authors:** Cara Monroe, Amy Lyons-Ketchum, Horvey Palacios, Justin Lund, John R. Walkup, Kent Smith, Stefan Wilhelm, Rajagopal Ramesh

**Affiliations:** 1 OU Health Stephenson Cancer Center, University of Oklahoma Health Campus, Oklahoma City, OK 73104, USA; 2 Department of Pathology, University of Oklahoma Health Campus, Oklahoma City, OK 73104, USA; 3 Graduate Program in Biomedical Sciences, University of Oklahoma Health Campus, Oklahoma City, OK 73104, USA; 4 Department of Anthropology, University of Oklahoma, Norman, OK 73109, USA; 5 Department of Anatomy and Cell Biology, Oklahoma State University Center for Health Science, Tulsa, OK 74107, USA; 6 Department of Anthropology, California State University, Fullerton, CA 92831, USA; 7 Stephenson School of Biomedical Engineering, University of Oklahoma, Norman, OK 73019, USA

**Keywords:** Cancer, Education, Training, High School, Native American, Indigenous, STEM, Oklahoma

## Abstract

Youth Enjoy Science (YES) Oklahoma (OK) Scholars is an eight-week summer program that engages American Indian high school students in cancer and biomedical research. YES OK uses a relational, place-informed educational model, with a curriculum that emphasizes (1) increasing cancer biology and public health knowledge, (2) developing laboratory and scientific communication skills, (3) strengthening college and career preparation, and (4) connecting scientific learning to cultural and community contexts. YES OK Scholars offers two university courses for general education credit and integrates on- campus lectures and laboratory research experiences, with virtual instruction to increase accessibility. Long-term sustainability is supported through near-peer and community networks, where current and former Scholars contribute to recruitment, mentorship, and community engagement. Implementing a place-informed curriculum posed logistical and institutional challenges; however, through ongoing program adjustments, partnership development, and new partnerships, YES OK developed an ethically transferable framework for increasing Indigenous student participation in cancer research. This paper reports outcomes and implementation challenges since program inception.

## Introduction

The National Cancer Institute’s Youth Enjoy Science (YES) Program is a National Institutes of Health funded initiative for students from middle school through the undergraduate level. This five-year program expands their pathways into cancer research through paid research experiences, mentorships, and community-based cancer and STEM education [1–3]. The YES Oklahoma (YES OK) program achieves this goal within the cultural, historical, and socioeconomic contexts of Tribal Nations in Oklahoma by instituting a relationship-driven, place-informed learning model that engages American Indian Junior and Senior high school students [4, 5]. The OU Health Stephenson Cancer Center (OU SCC) leads the program in partnership with the University of Oklahoma (OU), the Oklahoma State University Center for Health Science (OSU-CHS), and the OSU-College of Osteopathic Medicine (COM) at the Cherokee Nation. In this article, YES OK Scholars refers to the instructional component of YES OK.

Oklahoma is home to 38 federally recognized Tribal Nations [6]. American Indians in Oklahoma experience cancer incidence and mortality rates nearly 1.6 times higher than the national average [7]. Much of this disparity in cancer outcomes stems from inequitable access to screening and care along with limited healthcare literacy [8] — barriers that a stronger Indigenous healthcare, research, and public-health workforce could mitigate [8–10].

American Indian representation in higher education across the nation remains limited [11, 12]. As of 2024, American Indians and Alaska Natives represented only 0.3% of all doctoral recipients nationwide and only 0.1% of doctoral recipients in the biological and biomedical sciences [13]. However, research indicates that grounding learning environments in cultural contexts, tribal and family relationships, and local relevance strengthens persistence in higher education [6, 11, 12]. Guided by the Six Rs of Indigenous Research [13, 14] — *respect*, *relationship*, *representation*, *relevance*, *responsibility*, and *reciprocity* — YES OK integrates scientific training within a place-informed educational model that connects cancer research to students lived experiences and community contexts. In this article, we describe the cancer research experience of the Scholars, and outcomes on completion of the program.

## Methods

YES OK Scholars, selected through an application process and interview of applicants engage in an eight-week summer program to support professional development and advancement into biomedical research, public health, and cancer career pathways. The number of scholars selected varies and ranges from to seven to twelve Scholars per year. Scholars earn university credit through two concurrent enrollment courses that alternate each year: One introduces foundational concepts in cancer biology and laboratory skills, and the other examines cancer and public health in Oklahoma and Tribal communities. Both courses include applied mentored research experiences in which Scholars work alongside faculty, graduate researchers, research staff, and near-peer mentors.

During the first four weeks, Scholars reside on the University of Oklahoma campus in Norman, engaging in daily lectures and hands-on laboratory activities complemented by structured study hours, professional development workshops, and collaborative team-building events. The final four weeks are virtual, which maintains program goals while allowing Scholars to balance summer responsibilities. The curriculum emphasizes four core developmental objectives: (1) build conceptual knowledge of cancer biology and public health implications, (2) develop laboratory and scientific communication skills, (3) boost college and career preparation, and (4) connect scientific learning to cultural and community settings.

A multi-faceted mentorship model centered on peer-peer, near-peer, and mentor–mentee relationships supports continuity across program cohorts and instills a sense of belonging, with returning Scholars taking on expanded roles as near-peer mentors. Our program operates as a braided pathway [15] rather than a linear pipeline, with students entering from varied backgrounds, engaging in shared coursework and mentorship, and moving toward multiple possible next steps. Such steps could include returning to mentor new cohorts, performing health and education outreach, pursuing career-oriented training, or participating in undergraduate summer programs.

### Participation Logistics

YES OK Scholars was developed through consultation with Tribal partners and community stakeholders from Southwest Oklahoma, including representatives connected to the Comanche Nation, Kiowa Tribe, and Wichita and Affiliated Tribes. These consultations occurred through the Southwest Oklahoma STEM Alliance and included Native American teachers, school Indian Education programs, parents, and Tribal higher education representatives. Program design was intended to align with local priorities while also supporting students’ existing family and community responsibilities. These discussions indicated that many students could not commit to an uninterrupted eight-week on-campus program because of family responsibilities, cultural activities, summer employment, and transportation barriers, particularly for students from rural and geographically dispersed communities. In response, YES OK Scholars adopted a hybrid structure of four weeks on campus followed by four weeks of virtual instruction. Although the consultation process was rooted in Southwest Oklahoma, the resulting hybrid model continued to support students effectively as recruitment expanded to additional Tribal Nations across the state.

### Scholar Recruitment

Scholar recruitment is conducted through dissemination of program announcements via the Stephenson Cancer Center website, social media, printed flyers, and mass email outreach to schools and Tribal education entities. Recruitment is further supported through educational fairs, partner organizations, school counselor referrals, and word-of-mouth within American Indian communities and among YES Alumni and their families. The initial program design was informed through early consultation, and recruitment later expanded to reach students from additional Tribal Nations across Oklahoma.

### Pedagogical Framework

Table 1 illustrates how YES OK Scholars integrates research training within a place-informed educational approach guided by the Six Rs of Indigenous Research [13, 14]. These principles form a community-based participatory research design that emphasizes Tribal sovereignty, relational accountability, and shared benefit [16, 17]. The resulting program design:

- focuses its content on those cancer and public health issues disproportionately impacting Tribal populations in Oklahoma,
- integrates Scholars’ cultural histories, family networks, and lived experiences, and
- recognizes family knowledge and community relationships as meaningful sources of insight.

These principles are introduced early through a two-day workshop on Indigenous Research Ethics and Tribal Data Sovereignty. Scholars examine how Indigenous “ways of knowing” inform ethical research practice by critically comparing Tribal and institutional research priorities, including issues of sovereignty, consent, data governance, and community benefit. By analyzing hypothetical research proposals involving Tribal biological samples and role-based scenarios within Tribal healthcare systems, Scholars examine how resource constraints, community values, and external stakeholders shape research and health-care decisions.

## Results

To date, YES OK Scholars has enrolled 22 students representing 12 Tribal nations across Oklahoma (Caddo Nation, Cherokee Nation of Oklahoma, Cheyenne and Arapaho Tribes, Chickasaw Nation, Choctaw Nation of Oklahoma, Citizen Potawatomi Nation, Eastern Band of Cherokee Indians, Fort Sill Apache Tribe, Kiowa Tribe, Muscogee Creek Nation, Otoe-Missouria Tribe, and Quapaw Tribe). Program participation and retention remain strong with 96% of our Scholars completing the full 8-week program and, among those eligible, 100% returning for a second summer course and acting as near-peer mentors. Scholars also received awards, recognitions, and scholarship offers during and after high school, with cohort-level totals shown in Table 2.

Scholars completed surveys of health-related content knowledge and cancer awareness at the beginning and end of their summer involvement. As shown in Table 3, participants demonstrated gains in biology and public health knowledge including improved understanding of cancer risk factors, Tribal health, and Oklahoma cancer disparities. End-of-program interviews across cohorts indicate that YES OK Scholars supports career pathway development through three interrelated outcomes: (1) stronger sense of belonging in scientific spaces, (2) greater confidence in navigating college environments, and (3) deeper appreciation for the role that public health and medical research play in community well-being. Students reported increased confidence in discussing cancer research and treatment as well as improved understanding of multi-level cancer risk factors (individual, family, community, and Tribal) and Tribal public health resources. Based on course assignments and communication with students and their families, Scholars connected cancer biology and public health concepts to experiences within their own families, fostering conversations about cancer that had not previously occurred and supporting both academic learning and intergenerational dialogue on health.

### Challenges

Program implementation required navigating practical and institutional constraints, including housing and supervision policies for minors, concurrent enrollment, ACT/SAT eligibility requirements, and uneven academic preparation among Oklahoma schools with limited access to advanced coursework and standardized testing, where testing is often not required until students’ senior years. Despite strong student interest, these conditions complicated recruitment and enrollment.

YES OK Scholars addressed these constraints through ongoing program adjustment and partnership development and by providing college-level coursework and mentored research experiences that supplement limited advanced STEM offerings. Addressing institutional constraints required proactive and sustained collaboration within the university, as administrative structures, housing programs, and oversight of concurrent enrollment shifted over time. For example, the discontinuation of an initial summer housing option necessitated new partnerships with continuing education–based summer programs. Furthermore, changes in concurrent enrollment administration required coordination with new units and personnel. Concomitant collaborations with academic departments and campus support units (e.g., libraries, writing centers) strengthened mentorship and student professionalization, while external partnerships with a local community college expanded access to ACT preparation and additional testing opportunities.

## Discussion

The hybrid format of YES OK Scholars supports academic requirements while honoring family and community responsibilities, creating a shared learning environment where coursework, laboratory work, college preparedness, and career professionalization occur alongside peers, near-peer mentors, and faculty. These mentorship and cohort-based learning frameworks contribute to Scholars’ reported sense of belonging in scientific and university spaces. Through this approach, our Scholars have been able to successfully matriculate to colleges and universities across the county.

Participation in Indigenous research ethics and Tribal data sovereignty training further supported Scholars’ development of a professional identity toward collective accountability — a shift reflected in their community engagement after program completion. Returning Scholars mentor new cohorts and lead STEM and public health outreach efforts in their home communities. For example, one Scholar contributed voice recordings in their Indigenous language for public health posters disseminated within their Tribe. Cohort and community networks are further strengthened through cancer center–wide activities such as our high school youth programs and the Cancer Communication Awareness Challenge, which bring YES Scholars into shared learning spaces with our institutions undergraduate, graduate, and medical student programs exposing them to additional future biomedical training pathways.

## Conclusion

YES OK Scholars demonstrates that relationship-centered, place-informed cancer education can effectively support career pathway development for Indigenous students. Grounded in students’ lived experiences, the program integrates Indigenous research principles with academically rigorous biomedical training, preparing students to engage in cancer research and public health efforts in regions where cancer disparities persist. Its near-peer networking model supports long-term sustainability through cohort and community networks. While not intended as a generic template, YES OK Scholars offers career development programs in an ethically transferable framework when co-developed with the communities and partnerships it serves.

## Figures and Tables

**Table 1:** Alignment of the six Rs of indigenous research with YES OK program activities

R	Place-informed Principle	YES OK Practice
Respect	Learning recognizes students’ lived experiences, family contexts, and community networks as meaningful knowledge sources	Opening activities invite students to share places, people, or experiences that shape who they are. Program planning and consent processes are developed in collaboration with Tribal partners and community educators
Relationship	Knowledge develops from continuity, shared experiences, and trust	Layered mentorship networks connect Scholars with faculty, graduate trainees, undergraduate mentors, and returning Scholars across cohorts. Cancer center–wide and AI-focused initiatives (e.g., Cure Jr., Cancer Communication Awareness Challenge, Native Explorers, NARCH, GEN) further situate Scholars within multi-level learning networks spanning secondary, undergraduate, graduate, and medical training
Representation	Students benefit from seeing people with shared lived experiences in scientific and health fields	Indigenous and Indigenous-serving researchers, clinicians, and public health professionals serve as instructors, mentors, and guest speakers
Relevance	Learning connects to local health concerns and community realities	Coursework centers on cancer disparities and public health priorities specific to Oklahoma
Responsibility	Research is accountable to communities, not only individuals	Scholars complete a bioethics and Tribal data sovereignty workshop addressing consent, benefit, and data stewardship
Reciprocity	Learning involves sharing knowledge and supporting others	Scholars return knowledge through peer mentoring, STEM demonstrations, and youth or community outreach

**Table 2:** YES Oklahoma scholars outcomes by year

Cohort (Year)	Total Scholars	Juniors	Seniors	Female	Male	Returning Scholars Eligible from Prior Year (%)	Seniors Matriculating to College (Year)	Awards, Recognition, Scholarship (total count)^±^
Cohort 1 (2023)	7	2	5	6	1	0	5/5 (100%) enrolled in college (2024)	47
Cohort 2 (2024)	10	3	7	7	3	3 (100%)	7/7 (100%) enrolled in college (2025)	54
Cohort 3 (2025)	10	3	7	7	3	3 (100%)	Not yet eligible†	9

† Cohort 3 scholars were still in high school at the time of reporting

Returning scholars are Senior HS students who entered the program as Junior HS students in prior year ^**±**^Values reflect the total number of awards, recognitions, and scholarship offers identified for scholars in each cohort at the time of reporting; totals represent items received, not the number of students receiving them

**Table 3:** Survey results for Cohort 1–3, with 22/25 students reporting. Each score reflects a possible 100 points. The column titled “%Δ”refers to the percent change in results. Percent increase (+ %Δ) was calculated relative to pre-program scores and rounded to the nearest whole percent. Note this also includes data from returning students

Category	Pre-YES	Post-YES	%Δ
*Increased Knowledge*			
General biology	48	69	72%
Cell biology	47	74	57%
Cancer biology	32	78	144%
Biomedical Sciences	43	70	63%
Public health	48	75	56%
*Awareness of Cancer Incidence and Risk Factors*			
General cancer risk factors	47	81	72%
Family medical history	63	79	25%
Family history of cancer	54	77	43%
Native American cancer risk factors 35		77	120%
Tribal health services	39	76	95%

## Data Availability

The data is not publicly available as the number of participants is small and potentially identifiable.
